# Barriers to accessible ophthalmic imaging device uptake: an exploratory study of ophthalmology care provider perspectives

**DOI:** 10.1038/s41433-026-04751-3

**Published:** 2026-07-15

**Authors:** Julia R. Yamazaki-Tan, Alexandra I. Manta, Mark H. B. Radford

**Affiliations:** 1 Department of Ophthalmology Research, Queensland Eye Institute, Brisbane, QLD Australia; 2 Faculty of Health, Medicine and Behavioral Sciences, University of Queensland, Brisbane, QLD Australia; 3 Doctoral School of Medicine and Pharmacy, George E. Palade University of Medicine, Pharmacy, Science and Technology, Târgu Mureș, Romania; 4 Department of Ophthalmology, Princess Alexandra Hospital, Brisbane, QLD Australia; 5 School of Optometry and Visual Science, Faculty of Health, Queensland University of Technology, Brisbane, QLD Australia; 6 George E. Palade University of Medicine, Pharmacy, Science and Technology, Târgu Mureș, Romania

**Keywords:** Tomography, Technology

## Abstract

**Background and objectives:**

Clinical ophthalmology often depends on imaging to support accurate diagnoses. However, traditional tabletop imaging machines often fail to accommodate patients with mobility impairments. Despite this, few clinical settings routinely use accessible ophthalmic imaging devices to address these concerns. This study aims to identify perceived barriers to the uptake of accessible ophthalmic imaging devices to inform changes and improve patient care.

**Methods:**

In this exploratory mixed-methods survey study, a questionnaire was distributed to ophthalmology care providers across Australia from 2023 to 2024. Outcomes included perceived barriers to implementing accessible imaging equipment, perspectives on these devices and features improving accessibility.

**Results:**

The questionnaire returned 51 responses, equally distributed between public (51.3%, *n* = 20/39) and private (48.7%, *n* = 19/39) metropolitan healthcare settings. Participants included ophthalmic nurses (29.4%, *n* = 15/51), ophthalmology trainees (21.6%, *n* = 11/51), optometrists (17.6%, *n* = 9/51), orthoptists (13.7%, *n* = 7/51), ophthalmologists (7.8%, *n* = 4/51) and ophthalmic clinical assistants (9.8%, *n* = 5/51). Imaging was most difficult to provide for patients using wheelchairs (39.5%, *n* = 15/38). While most practitioners (74.4%, *n* = 29/39) were confident that accessible devices returned reliable images, 48.7% (*n* = 19/39) identified cost as a barrier, and 39.2% (*n* = 20/51) believed accessible devices were still difficult to use in patients with disabilities. Respondents reported that better design of tabletop-mounted systems, adaptability, training and funding would improve uptake and use of accessible devices.

**Conclusions:**

Accessibility for patients with mobility impairments needs further development. Intuitive design and access to funding may be key to improving the quality of care. Further studies should focus on patient perspectives and specific design features.

## Introduction

Mobility impairments are known to complicate radiographic examination and other types of medical imaging and these difficulties may degrade the quality of ophthalmic care and clinical outcomes for patients with mobility impairments. A 2009 focus-group study of 75 individuals with physical or sensory disabilities revealed that many experience difficulty and distress while interacting with imaging devices that fail to accommodate a diverse range of mobility impairments [1]. One patient who uses a wheelchair described their experience with ophthalmic imaging as ‘uncomfortable,’ with staff needing to ‘push [their] back up, in order to touch [their] chin to the machine,’ exemplifying one of the most common challenges associated with accessibility and patient comfort during diagnostic procedures [1].

While literature on the impact of accessibility concerns on ophthalmic imaging is lacking, patient concerns about *non-ophthalmic settings* include a lack of height adjustment options, difficulty aligning wheelchairs with equipment and difficulty with staff who were unwilling to make supportive accommodations [1]. Medical imaging staff themselves may have concerns about liability, lack of training on accommodation of mobility aids, lack of devices or slings required for safe transfer and may require extra time or staff to perform imaging for patients with mobility impairments [2–5]. Challenges like these could contribute to inaccurate diagnostic data in radiographs, mammography and computed tomography imaging studies [6, 7]. Consequently, patients with mobility impairments are often unable to receive truly equitable health services, which contributes to psychological distress and the continued marginalisation of individuals with disability [4, 8]. Similar challenges could contribute to inaccurate or low-confidence diagnosis and treatment in ophthalmic settings, also potentially creating poorer outcomes for patients with disabilities.

Other factors intersect with and interact with mobility impairment, including age-related disability and dementia and obesity. Australia’s population is experiencing demographic aging, which is associated with declining mobility and a heightened dependence on mobility aids, as well as increasing prevalence of dementia [9–11]. The Australian Bureau of Statistics Report on Disability, Ageing and Carers (2022) revealed that 52.3% of Australians aged over 65 have a disability [12]. Furthermore, amongst those living with a disability, 18.4% use aids for mobility, although it is not known how this percentage changes across age groups [12]. Dementia is also known to complicate radiographic imaging, but it is yet unknown how this impacts ophthalmic imaging modalities [13]. Another key factor is obesity, which affects 31.7% of Australian adults [14] and which may complicate radiologic imaging via technological limitations, weight limits and positioning difficulties [3, 6]. As the Australian population ages and the prevalence of mobility impairments and obesity increases, ensuring the accessibility of ophthalmic imaging becomes not just an issue of convenience, but a public health necessity.

Some devices already exist that may help address these physical accessibility needs. For the purposes of this report, ‘accessible imaging devices’ include those with specific features enhancing portability (including handheld tools), greater adjustability or range of positioning (including height adjustments or headsets) and spatial compatibility with wheelchairs and other mobility aids. Several reports detail the design and early use of handheld devices for retinal imaging in children or patients restricted to a supine position [15–17]. Virtual-reality headsets are now used for automated perimetry tests, although some concerns about their accuracy remain [18–20]. Some mainstream devices also have accessibility features, including handles on tabletops for patient use, wide foot bases for wheelchair access, adjustable table heights and removable chinrests. However, these devices are not yet standard in most Australian ophthalmology clinics and the data on their adoption is sparse. A qualitative study of physicians in the USA revealed that major barriers to improving the physical accessibility of examination equipment were high costs, limited space and inadequate compensation for the additional time, effort and purchase of specialised equipment needed to care for patients with mobility impairments [21].

Few studies examine the specific barriers to implementation of accessible devices in Australian clinical practice. This study aims to address this gap and determine what factors prevent accessible imaging machinery from being widely used in ophthalmic healthcare settings and what changes could increase uptake.

## Methods

A survey was distributed to a convenience sample of 51 ophthalmology healthcare workers in Australia from April 2023 to December 2024. This included providers from public and private practice, clinic-based and hospital-based work and metropolitan and remote work. Healthcare workers eligible for inclusion are practicing ophthalmologists, ophthalmology registrars, optometrists, orthoptists, ophthalmic nurses or ophthalmic clinical assistants. The survey was distributed both digitally via email and the RANZCO Survey webpage and in-person by one of the study authors. Reminders were not sent. Several university optometry departments across Queensland, New South Wales, Victoria, South Australia, the Australian Capital Territory and Western Australia were contacted by phone and email, but no responses were received via this pathway. There was no relevant university department of optometry in the Northern Territory or Tasmania, hence the lack of contact through this direct method in these regions. Due to the method of survey distribution, the number and characteristics of non-responders were not calculated; thus, the response rate was not calculated. The decision to use a convenience sampling method reflects the exploratory intent of this study, but lends itself towards the introduction of sampling biases.

A combination of qualitative and quantitative data was sourced using a de-identified questionnaire. The questionnaire was briefly piloted with optometrists and ophthalmologists from a private ophthalmology clinic in metropolitan Queensland. The survey was not validated nor derived from existing instruments, surveys or tools. The items written for the survey were informed by current physical accessibility challenges described in the literature on ophthalmic and non-ophthalmic imaging and an open-response section was included to identify additional experiences not hitherto mentioned in the literature. The questionnaire consisted of thirty-one items across four sections. The first section assessed respondent demographics. The second section used a 5-point Likert scale where participants indicated how significant an obstruction each listed barrier poses to implementation. An example prompt is: ‘Accessible imaging devices cost too much’. The third section contained multiple-choice questions regarding experiences with disability and imaging. The final section included open-answer questions to prompt participants to report any other factors that were not included in the questionnaire and to provide recommendations for the implementation of accessible imaging devices.

The primary outcome, sourced from section two, was the perceived significance of key barriers to the implementation of accessible imaging equipment. Secondary outcomes, sourced from sections three and four, included any personal experiences with disability, implementing accessible imaging devices and any recommendations proposed to increase uptake of accessible devices.

The Likert-scale item data were described using the percentage endorsement of each statement, which was defined as the percentage of participants answering ‘agree’ or ‘strongly agree’. In addition, the percentage of respondents selecting each point on the 5-point Likert scale was reported per item. Section three data was reported as a percentage of respondents reporting each answer. Data from the fourth phase of the questionnaire were analysed via inductive thematic analysis. One researcher (JYT) coded the data and Microsoft Excel [22] was used to assign codes and organise these into themes.

Ethics approval was obtained from the Royal Australian and New Zealand College of Ophthalmologists Human Research and Ethics Committee (Reference 153.22).

## Results

**Fig. 1:**
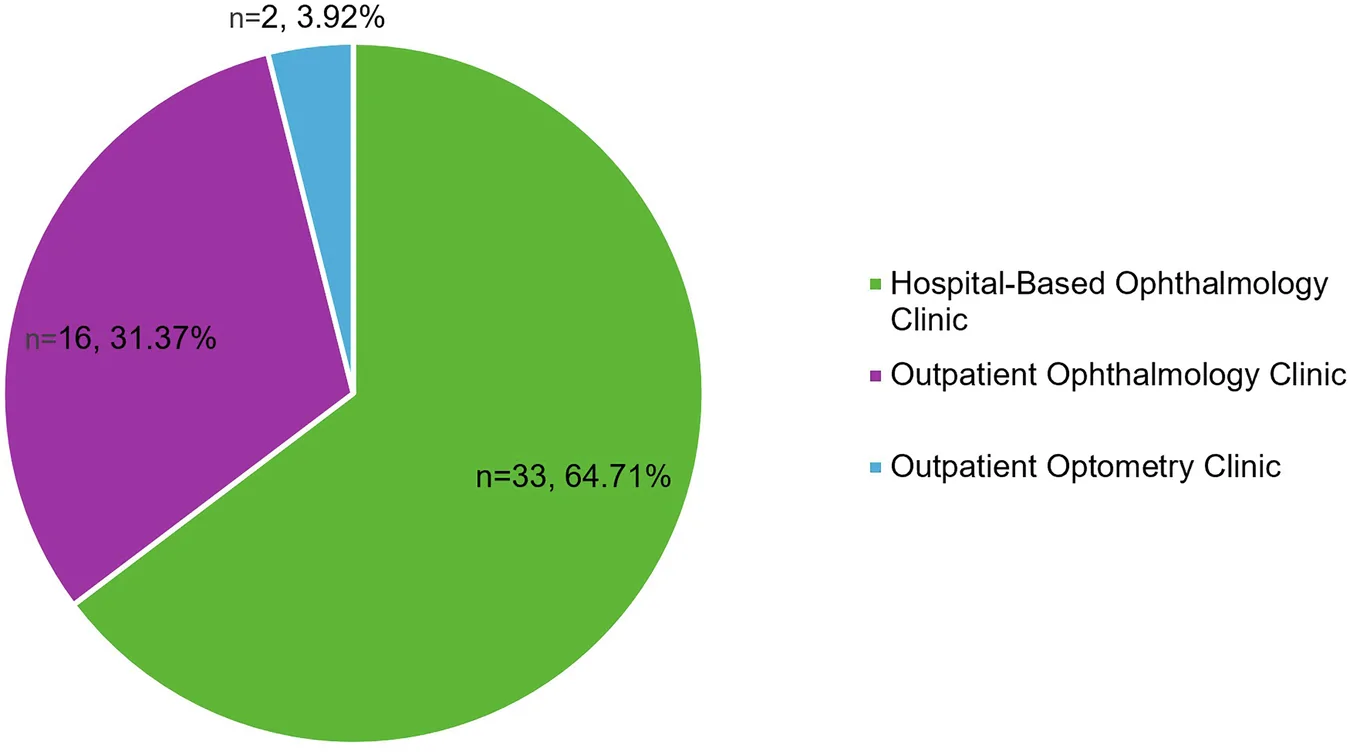
Proportions of participants primarily practicing in different types of clinical settings. Data was available for all participants for this question.

**Fig. 2:**
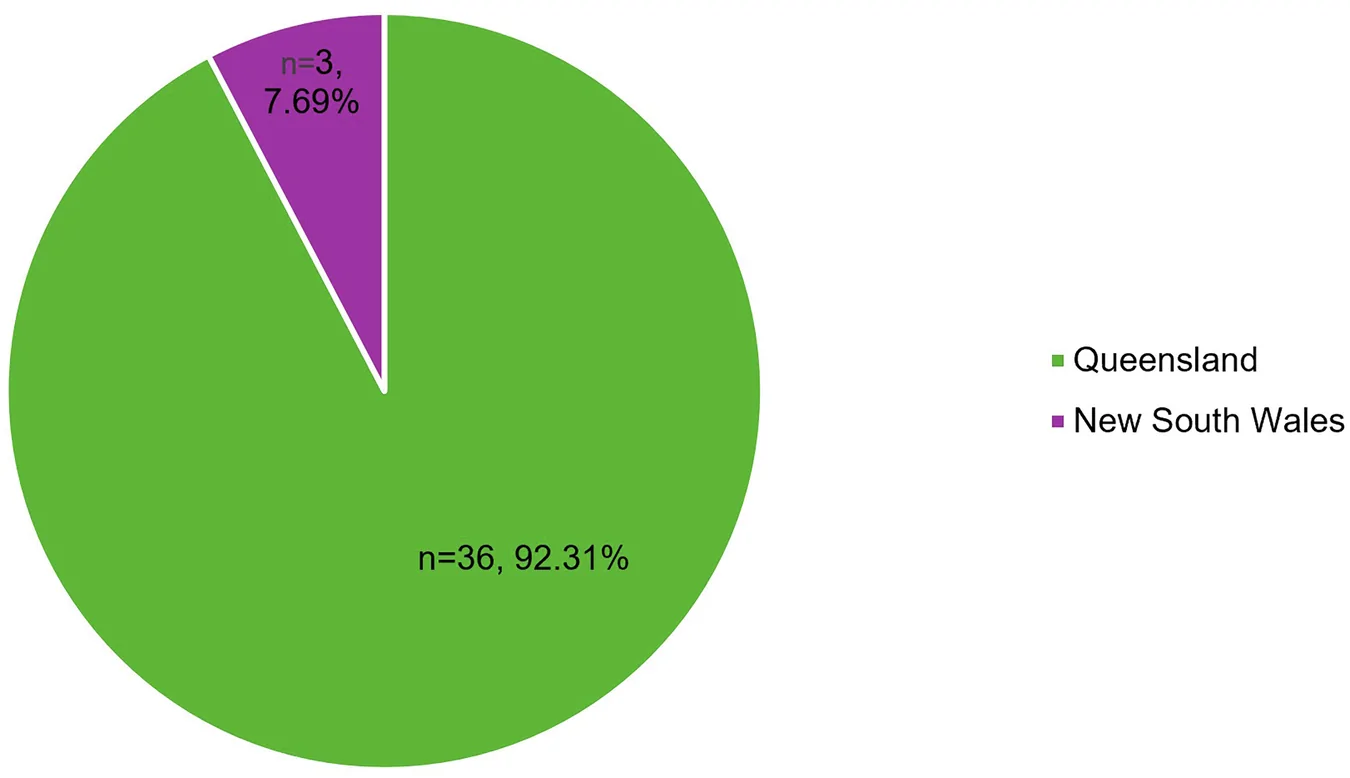
Proportions of participants primarily practicing in each Australian state. Data was available for 76.5% of all participants for this question.

**Fig. 3:**
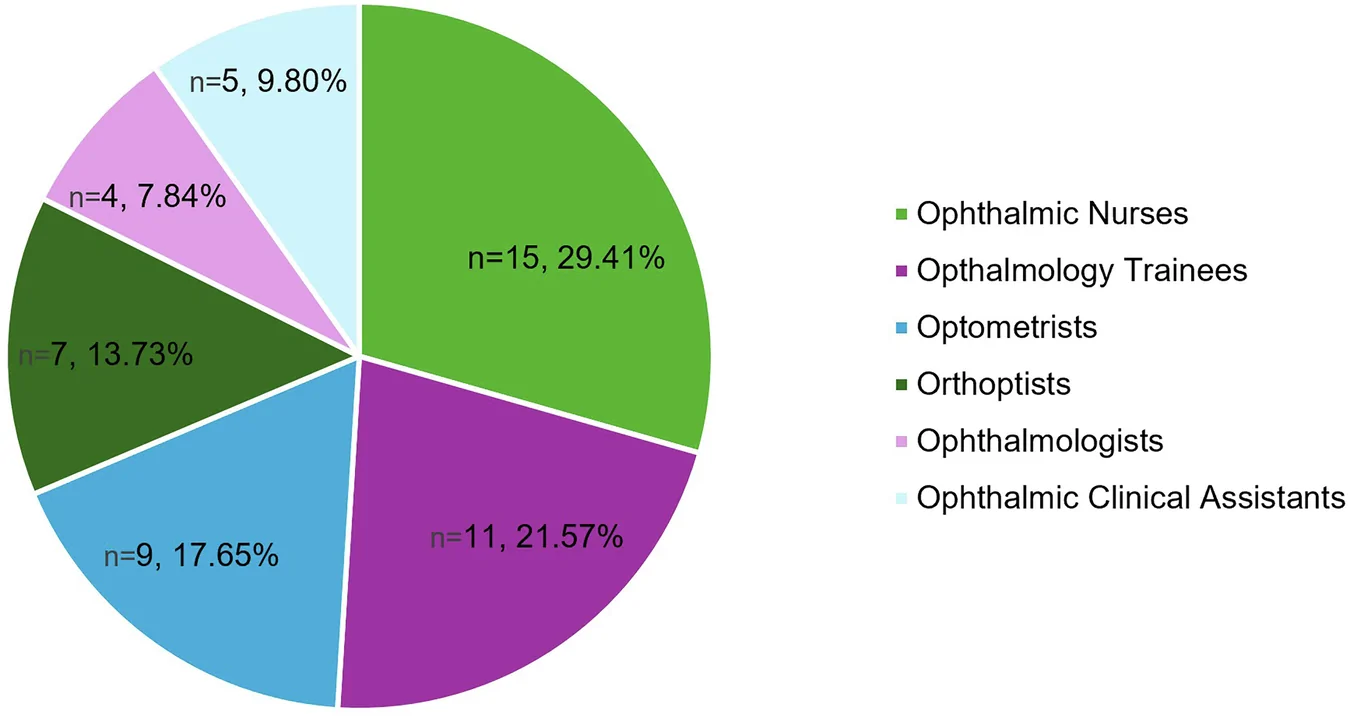
Clinical roles of survey respondents. Data was available for all participants for this question.

The survey received 51 responses after 20 months. This was due to low uptake of the survey and an extension of the intended survey period (from April 2024 to December 2024) to encourage more responses. Nevertheless, this was regarded as a significantly low sample size. Informed consent was obtained from all subjects. Twelve questionnaires contained missing data points, which were interpreted as ‘no response provided’. Amongst the surveys completed on paper (*n* = 21), some questions were systematically unanswered. While the cause was unknown, the systematic nature suggested an error in printing or failure to fill out both sides of each paper sheet. Consequently, the percentage endorsement for each item was calculated based on the total number of responses received. Inferential statistics and subgroup analyses were unable to be performed due to the low volume of responses and missing data points. Responses were derived evenly from public (51.3%, *n* = 20/39) and private (48.7%, *n* = 19/39) metropolitan healthcare settings. Most respondents worked in hospital settings (64.7%, *n* = 33/51), followed by ophthalmology clinics (31.4%, *n* = 16/51) and optometry offices (3.9%, *n* = 2/51) (Fig. 1). Most participants were based in Queensland, Australia (92.3%, *n* = 36/39) and a minority from New South Wales, Australia (7.7%, *n* = 3/39) (Fig. 2). Respondents included ophthalmic nurses (29.4%, *n* = 15/51), ophthalmology trainees (21.6%, *n* = 11/51), optometrists (17.6%, *n* = 9/51) orthoptists (13.7%, *n* = 7/51), ophthalmologists (7.8%, *n* = 4/51) and ophthalmic clinical assistants (9.8%, *n* = 5/51) (Fig. 3).

Participants reported that imaging was most difficult in patients using wheelchairs (39.5%, *n* = 15/38) or those living with intellectual disability or dementia (34.2%, *n* = 13/38). Approximately half (51.3%, *n* = 19/38) of respondents indicated that wheelchair use was the most frequently encountered disability in their clinical setting. Most respondents agreed that their clinic already uses a device that can image patients with disabilities, even if with difficulty (69.2%, *n* = 27/39). Nearly half of respondents agreed that their clinic is not familiar with the accessible imaging devices available (47.1%, *n* = 24/51). Fewer respondents reported that their clinic lacked sufficient staff to operate accessible imaging devices (21.1%, *n* = 8/38). Some respondents agreed that their clinic was too busy to organise the purchase, set-up and training on accessible imaging devices (29.4%, *n* = 15/51). A minority of respondents believed that their clinic does not see a large population of patients with disabilities (17.6%, *n* = 9/51). Participants reported use of various accessible imaging devices in their clinical settings, including handheld OCT probes, handheld autorefractors, handheld slit-lamps, visual fields headsets, handheld tonometers, contact axial length scans and portable B-scans.

**Fig. 4:**
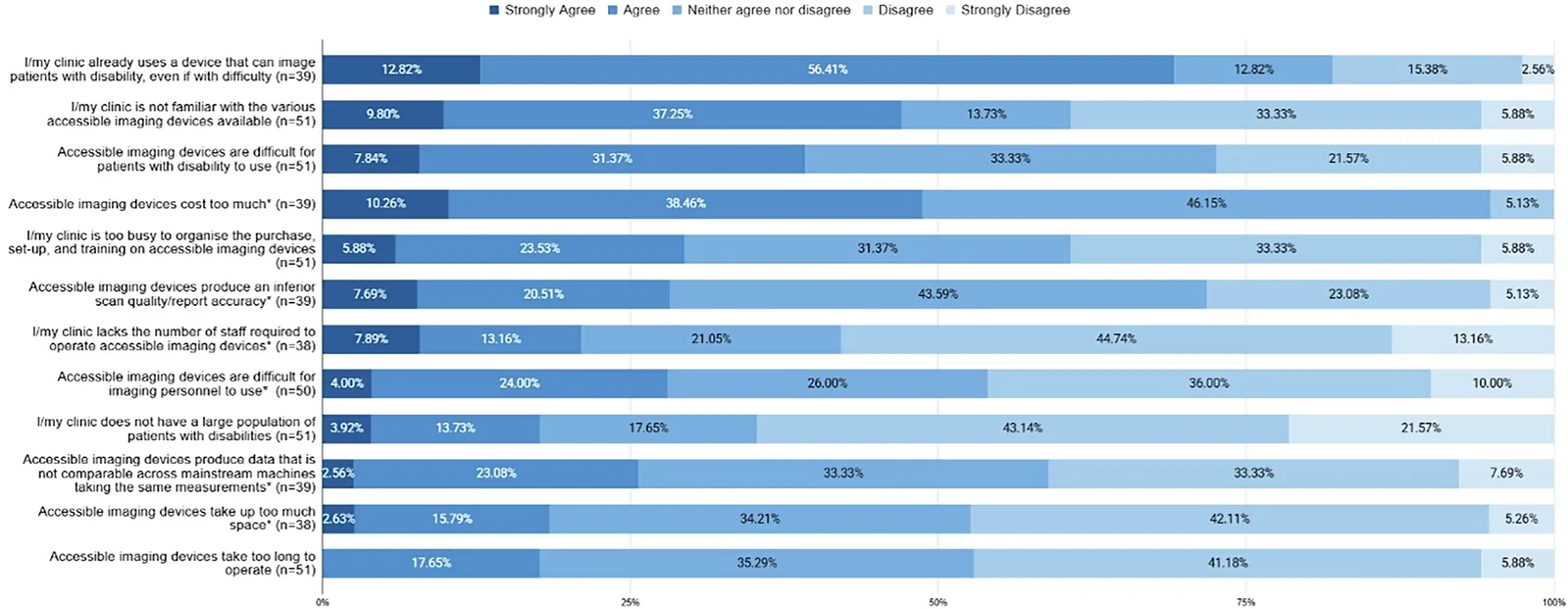
Endorsement of beliefs about device factors and clinic factors influencing use or uptake of accessible imaging devices. Participants indicated their level of agreement with several statements about accessible imaging devices. An asterisk (*) indicates questions where responses were missing, either due to participants opting not to answer or due to a recording error. Total number of respondents indicated for each question.

Some participants agreed that accessible imaging devices are difficult for patients with disability to use (39.2%, *n* = 20/51) (Fig. 4). A smaller group endorsed that accessible imaging devices are difficult for imaging personnel to use (28.0%, *n* = 14/50). Approximately half of the respondents agreed that accessible imaging devices cost too much (48.7%, *n* = 19/39). Few participants agreed that accessible imaging devices produce an inferior scan quality or report accuracy (28.2%, *n* = 11/39); a similar number agreed that accessible imaging devices produce data that is not comparable across mainstream devices taking the same measurements (25.7%, *n* = 10/39). A small few endorsed the belief that accessible imaging devices take up too much space (15.8%, *n* = 6/38) and that accessible imaging devices take too long to operate (17.6%, *n* = 9/51).

In the open-response section, participants described a variety of device-related and systemic or organisational factors they believe influence the accessibility and reliability of accessible imaging devices. Key points were grouped into themes (Table 1).

**Table 1:** Illustrative quotes from each key theme.

Theme	Key Codes	Illustrative Quotes
Device and software-related accessibility features	Desired functionalities and use cases for an ideal device	‘The ability to use [accessible devices] with a patient in a wheelchair.’ ‘If the devices mounted to tables can have the head-mount angle adjustable ‘Ability to adapt devices for individual needs.’
’	Choice of imaging table and positioning of devices on imaging table	‘A table that can be lowered to adjust for wheelchair height, most instrument tables don’t go low enough.’ When choosing slit lamp tables, think about whether the wheels might fit underneath the table. Also, for obese patients, whether the slit lamp is mounted to the edge of the table or as close as possible.’ ‘HVF on a slide out table to bring closer towards the patient with neck troubles rather than having the patient lean forward.’ ‘Notable experiences such as using the OCT on a wheelchair bound patient. Barrier encountered due to the OCT table, unable to move it sideways to create room for the wheelchair.
	Medical chair with adjustable dimensions and stabilisation in tandem with regular or accessible imaging devices to produce stability and correct positioning	‘Velar medical chairs to more easily adjust the patients to correct positioning whilst being stable on a lockable chair.’
	Equipment with dual or multiple imaging capabilities saves positioning time	‘Optos with inbuilt OCT would be ideal rather than having to set patients up twice on different modalities.’
	Manual capture options	‘Manual capture/manual focus of an OCT scan if the patient is not in an optimal position (i.e., forehead not touching) but would still be able to acquire a decent scan’
	Portability	‘Easily moved between clinic rooms (i.e. mobile & compact).’
	Integration of data into the same review software	Ideally, companies with established footholds and software within the industry would market devices (or allow their software to integrate other devices) and data into their interpretations and review software.
	Option to remove unnecessary sensory stimuli for patients with global developmental delay or sensory sensitivities	‘When capturing images/scans for children with intellectual disabilities/global development delay/autism, having the option to remove sensory triggers like loud sounds and flashing lights would be very useful.’
Financial affordability, funding and incentives	Cost as a key barrier	‘Mostly the barriers have been approval for cost.’ ‘Cost of handheld slit lamps.’ ‘Low numbers per clinic and therefore difficult to justify the cost.’
	Need for government funding	‘Government funding should be available to purchase accessible imaging devices’ ‘Government priorities, policies and funding which could help healthcare facilities to invest in better technology.’
	Changes to device insurance policies	‘Extending insurance program, coverage to include accessible devices & reduce financial cost.’
	Organisational changes in understanding	‘Deeper understanding from staff about the need for accessible devices, understanding individual needs and appropriate language to support disability and accessibility.’
	Requirement for research to substantiate funding changes	‘Research to highlight that this group is often disadvantaged, as without accessible devices, they cannot be scanned effectively’ ‘Research into how many patients would require this.’
Training and familiarity	Increase the frequency of use in clinics to increase staff exposure and practice	‘Use in standard clinic for staff exposure to device capability.’
	Formal staff education and training	‘Training for imaging/assessing these patient populations and awareness of what’s available to help.’ ‘Educational sessions, I’m unsure of the general optometry practice’s knowledge of the existence of this equipment (e.g., VR VF testing).’ ‘Allocating an appropriate time for upskilling.’
	Reducing the need to ‘double up’ on equipment and training	‘The understanding that accessible devices could be used by everyone (this would be applicable to at least some equipment), so the practice/clinic wouldn’t necessarily need to double up on equipment.’
	Device technology representatives promoting the use of accessible technologies	‘Regular and engaged [device representative] presence to educate and train staff.’ ‘Demo machines to try. Training of staff to use.’

These are derived from the fourth portion of the questionnaire, where participants responded using free-form answers to describe their experiences, challenges and recommendations.

### Theme 1: device and software-related accessibility features

A key theme that arose was the need for adjustable equipment to accommodate individual needs. This included adjustable table heights, headrest angles, tools for stabilisation during patient movement and light, compact, portable or wearable equipment for use in positions comfortable to the patient. Participants specifically reported a need for machines that adapt to individual needs, including for patients with obesity or those using a wheelchair. It was suggested that machines mounted closer to the edge of the table may better serve patients with obesity and wider footrests were proposed to allow for wheelchairs to fully fit under the imaging table. Others noted the possibility of using specialised medical chairs with adjustable dimensions and stabilisation in tandem with regular or accessible imaging devices to produce stability and correct positioning. Some participants reported that equipment with dual or multiple imaging capabilities saves positioning time. Other, less-reported, but unique suggestions included: manual capture options, portability and the ability to integrate data from multiple devices into the same review and interpretation software and the option to remove unnecessary sensory stimuli for patients with global developmental delay or sensory sensitivities.

### Theme 2: financial affordability, funding and incentives

Another key concern reported in this section was the affordability of these machines. Several participants reported concerns about high costs, low budgets and low incentives. Corresponding with the previously endorsed concerns around cost, many participants reported a need for government funding for accessible devices and the associated insurance costs. Some participants also called for further research into the demand for accessible imaging and the disadvantages that patients with disabilities currently face, to incentivize purchase and provide an evidence base for government funding programs.

### Theme 3: training and familiarity

Another key need was staff training and familiarity with these machines. One suggestion was to increase the frequency of use in clinics to increase staff exposure and practice with these devices. Other participants noted that formal staff training would increase their capacity to work with patients with disabilities, understand their needs and communicate appropriately. Another participant noted that accessible, portable or handheld devices could be used on every patient, reducing the need to ‘double up’ on equipment and training, thus also saving costs and clinic space. Some participants reported that there could be a role for device technology representatives in promoting the use of accessible technologies through demonstrations.

## Discussion

Ophthalmology care providers report that they serve a significant population of patients with disabilities and most reported the use of devices that at least marginally meet the needs of patients with disability. There was a range of reported device-related and clinic-related barriers to providing appropriate devices, including unfamiliarity with device options, difficulty of patient use, training requirements and cost. The current study revealed mainly positive attitudes about the technical capabilities of accessible imaging technologies, but identified the need for adjustable physical elements of equipment and further organisational and systemic level supports to adopt these devices.

The findings of the current study align with current research indicating that cost is a major hurdle to improving accessibility of imaging equipment [21]. This may result from the high price of specialised imaging equipment, the fact that patients with disabilities comprise potentially a smaller subset of the total patient population and that any machines acquired specifically to image patients with mobility impairments will receive less use and are therefore more expensive relative to their level of productivity. Current research on imaging in patients with mobility impairments continues to be limited, it is unknown how other findings compare to pre-existing evidence. The results of this study begin to fill a gap in research literature on experiences of ophthalmology care providers with accessible imaging devices.

Practically, the findings of this study suggest that including accessibility modifications during the design process to improve ease of use may encourage greater uptake by clinics. In addition, device technology representatives have a key role in promoting the use of accessible, flexible and portable device options through demonstrations and staff training. Further, policymakers could consider providing financial incentives or subsidies for clinics that adopt such technologies or subsidizing the cost of re-training on new devices.

While current literature on accessible imaging technologies is limited, there are several anticipated benefits of adopting accessible imaging technologies. Overall, improving accessibility in ophthalmic imaging could lead to more inclusive care and greater clinical outcomes for patients with mobility impairments, potentially allowing for earlier diagnosis of conditions such as glaucoma or age-related macular degeneration, whose detection heavily relies on imaging data [23, 24]. While evidence is limited, adoption of accessible imaging devices may be one way to provide timely and accurate data in patients with mobility impairment, contributing to appropriate and timely treatment and reduced risk of misdiagnosis. Devices with accessible features are also more likely to accommodate a wider range of needs, regardless of the level of mobility impairment. This includes elderly or paediatric patients, patients of short stature, with temporary injuries or anxiety surrounding medical imaging devices. Appropriate imaging technologies may increase patient satisfaction and contribute to feelings of dignity, trust and belonging, especially for those who are chronically marginalised. From a healthcare system's point of view, more tailored imaging devices may decrease the staff time cost involved in assisting with imaging and boost financial and time efficiency on a clinic level.

In Australia, facilities providing diagnostic imaging were previously required to adhere to the Diagnostic Imaging Accreditation Scheme [25] to provide Medicare-funded services. These standards are now in the process of being superseded by the National Safety and Quality Medical Imaging Standards [26]. This new quality framework requires that medical imaging providers provide access to ‘an environment, facilities, equipment and devices that are fit for purpose, well-maintained and meet the needs of patients, including those with a disability…’ and that this care environment should be culturally safe and inclusive for individuals with a disability [26]. Managing physical accessibility concerns would fall under this requirement. Thus, the importance of accommodating patients with mobility impairments is not only a healthcare equity issue, but also a healthcare quality and regulatory issue.

The most salient limitation of this study is the low sample size, which increases the risk of sampling bias and random variability in the results, ultimately affecting the reliability of the findings. Moreover, the use of convenience sampling introduced significant sampling bias. Many participants were recruited from the same workplace settings and the sample was non-representative of ophthalmology care providers in the sampled regions. This may amplify findings relevant to certain clinical settings or workplaces over others. Some degree of response bias is also expected. For example, respondents from well-staffed clinics with little to no time pressures are potentially more likely to return the survey and less likely to endorse time constraints as a barrier to integrating accessible imaging devices. Further to this, systematically missing data in the returned surveys suggests that there may be underrepresentation of perspectives from participants who completed paper surveys, potentially due to a printing issue. Another key limitation of this study is its geographic focus on metropolitan regions of both Queensland, Australia and New South Wales, Australia. These regions have distinct demographic and service environments from other states. New South Wales has the highest rate of ophthalmologist coverage in Australia, compared to Western Australia and the Australian Capital Territory, which have the lowest coverage of ophthalmologists. Western Australia in particular relies heavily on telehealth ophthalmology services at a disproportionate rate to other states; this is largely due to a large rural area, with low specialist eyecare service coverage [27]. Both of these states and their health service environments were unrepresented in survey responses. As a result, the findings of this study may not be generalisable to the Australian population broadly. Furthermore, the sample was predominantly metropolitan, which is a very distinct healthcare service landscape compared to the rural or remote locations where the prevalence of disability is greater, but the ophthalmology workforce is sparser [28, 29]. Thus, specific rural and remote accessibility challenges were most likely unrepresented in this study, despite their relative importance and conclusions about rural ophthalmology settings are limited for this reason. More balanced coverage of Australian geographical regions and rural statuses could strengthen the relevance of future findings. Australian ophthalmology care is primarily delivered by optometrists, orthoptists, nurses, ophthalmologists and ophthalmology trainees, healthcare students and junior doctors. However, only 7.8% of the sample were ophthalmologists, which suggests underrepresentation of these individuals, who are often responsible for imaging equipment purchases. Furthermore, the largest occupational group within the current sample was comprised of nurses, followed by ophthalmology trainees. This may limit the generalisability of the findings to other healthcare systems with a different model of care, such as outpatient optometry clinics or non-teaching hospitals or those primarily staffed by optometrists or orthoptists. Further to this, the current study is limited by its focus on healthcare provider perspectives, which does not adequately represent the range of patient experiences or the limitations of device developers.

The findings of this study may inform key stakeholders involved in the design, development, purchase and funding of accessible ophthalmic imaging devices. Relevant stakeholders include medical technology engineers and manufacturers, government health departments, ophthalmology healthcare workers, clinic managers and patients themselves.

Future research could investigate the perspectives of patients with mobility impairments on their experiences receiving ophthalmic medical imaging, using surveys or focus groups. Another topic deserving investigation is the outcomes associated with accessible imaging device use in patients with mobility impairments. Future studies may compare scan quality, patient satisfaction and clinical outcomes for patients with mobility impairments between clinics that do and do not integrate accessible imaging technologies into their daily practice. There are several ways to increase clinic accessibility and patient satisfaction alongside changes in device design. Further investigations may focus on the utility of staff training programs organisational changes, such as extended appointment times and additional staffing for complex imaging or quiet, separate imaging areas for complex or difficult imaging.

## Conclusions

These exploratory findings suggest a need for accessibility features in ophthalmic imaging devices, as well as increased training and awareness surrounding accessible ophthalmic imaging devices, both at the design stage and during their clinical use and policy-level changes to support the adoption of these technologies in clinical practice. Improvements at each of these levels may be required to better serve patients with disabilities. Prioritising accessibility in ophthalmic care aligns with medical imaging standards of care, as well as broader efforts to reduce healthcare disparities and ensure that all patients, regardless of physical ability, receive quality care. The current study is limited in its generalisability across diverse healthcare settings and Australian geographic regions, underscoring the need for further and more substantial investigations into this topic.

## Summary

### What is known about this before

- Traditional tabletop ophthalmic imaging devices often fail to accommodate patients with mobility impairments.
- Despite this, few clinical settings routinely use accessible ophthalmic imaging devices to address these concerns.
- There is limited literature on which device, institutional and systemic factors increase the uptake of more accessible imaging devices in these settings.

### What this study adds

- Accessibility for wheelchair users and patients with intellectual disabilities, dementia and neurodiversity needs further development.
- Intuitive design and government funding may be key to improving the quality of care.
- Further studies should focus on patient perspectives and the required specific design features.

## Data Availability

The datasets generated during and/or analysed during the current study are available from the corresponding author on reasonable request.
